# Altered TRPM3‐Dependent Cytosolic and Mitochondrial Calcium Influx in Natural Killer Cells of Post‐COVID‐19 Condition Patients

**DOI:** 10.1002/eji.70240

**Published:** 2026-07-22

**Authors:** Chandi Tabeth Magawa, Natalie Eaton‐Fitch, Katsuhiko Muraki, Sonya Marshall‐Gradisnik

**Affiliations:** ^1^ National Centre for Neuroimmunology and Emerging Diseases, Health Group Griffith University, Gold Coast Campus Gold Coast Australia; ^2^ Consortium Health International For Myalgic Encephalomyelitis Griffith University, Gold Coast Campus Gold Coast Australia; ^3^ School of Pharmacy and Medical Sciences Griffith University, Gold Coast Campus Gold Coast Australia; ^4^ Laboratory of Cellular Pharmacology, School of Pharmacy Aichi‐Gakuin University Nagoya Japan

**Keywords:** calcium signalling, mitochondria, natural killer cells, post‐COVID‐19 condition, TRP channels, TRPM3

## Abstract

According to the World Health Organization (WHO), approximately 6% of COVID‐19 cases develop serious long‐term sequelae referred to as post‐COVID‐19 condition (PCC). Immunological disturbances such as persistent activation of immune cells and reduced cytotoxicity by natural killer (NK) cells are reported as key aspects in PCC. Recently, electrophysiological studies by our group demonstrated impairment of transient receptor potential melastatin 3 (TRPM3) ion channels in NK cells from PCC patients. The significant reduction in TRPM3 channel function and reduced functional activity by NK cells warrants further investigation. Hence, using live cell calcium (Ca^2+^) imaging ex vivo, we examined the downstream impact of TRPM3 ion channel dysfunction on intracellular and mitochondrial Ca^2+^ mobilization in NK cells from *N* = 8 PCC patients, age and sex matched to *N* = 8 PCC healthy controls (HC). Our findings provide new evidence of altered passive and TRPM3‐mediated Ca^2+^ influx, significantly impacting cytoplasmic and mitochondrial Ca^2+^ mobilization in PCC. Passive cytosolic Ca^2+^ influx amplitude (*p* < 0.0001) was significantly reduced in PCC; however, passive mitochondrial Ca^2+^ mobilization (*p* < 0.0001) was significantly increased. Importantly, cytoplasmic and mitochondrial response rates (slope, *p* < 0.001) to pregnenolone sulphate stimulation were significantly reduced in PCC. Consequently, TRPM3‐dependent cytosolic (*p* < 0.001) and mitochondrial (*p* < 0.0005) Ca^2+^ mobilization were significantly reduced in PCC compared with HC. Altered ion channel Ca^2+^ signalling can severely impact both the immune system and bioenergetic processes, potentially leading to broader systemic dysregulations underpinning the pathomechanism of the PCC condition, and warrants further investigations.

## Graphical abstract

Immunological disturbances such as persistent activation of immune cells and reduced cytotoxicity by natural killer (NK) cells are reported as key aspects in post‐COVID‐19 condition (PCC). Our findings provide new evidence of altered passive and TRPM3‐mediated Ca^2+^ influx, significantly impacting cytoplasmic and mitochondrial Ca^2+^ mobilization in PCC.

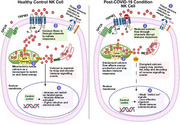

**Abbreviations**
ATP: adenosine triphosphate
BMI: body mass index
Ca^2+^
: calcium
CaM: calmodulin
CNS: central nervous system
COVID‐19: coronavirus disease 2019
CRAC: calcium‐release‐activated channel
ER/SR: endoplasmic/sarcoplasmic reticulum
F0: minimum fluorescence
FBC: full blood count
FCCP: carbonyl cyanide 4‐(trifluoromethoxy)phenylhydrazone
Fmax: fluorescence at maximum peak
HC: healthy control
MCC: Manders’ colocalization coefficients
ME/CFS: myalgic encephalomyelitis/chronic fatigue syndrome
*N*
: number of participants
NCNED: National Centre for Neuroimmunology and Emerging Diseases
NK cells: natural killer cells
PCC: post‐COVID‐19 condition
Pearson's *r*
: Pearson's correlation coefficient
PregS: pregnenolone sulfate
RFI: relative fluorescence intensity
ROI: region of interest
SARS‐CoV‐2: severe acute respiratory syndrome coronavirus 2
SD: standard deviation
SERCA: sarco/endoplasmic reticulum calcium‐ATPase
SF‐36: 36‐item short‐form health survey
SOCE: store operated Ca^2+^ entry
SPSS: statistical package for the social sciences
Stim1: stromal interaction molecule 1
*T*_1_
: time at minimum fluorescence intensity
*T*_1/2_
: half‐time to peak Ca^2+^ influx
*T*_2_
: time at maximum fluorescence intensity
TRP: transient receptor potential
TRPM3: transient receptor potential melastatin 3
WHO: World Health Organization

## 1 Background

The repercussions of the coronavirus disease 2019 (COVID‐19) caused by the severe acute respiratory syndrome coronavirus 2 (SARS‐CoV‐2) remain a global public health concern [1, 2]. Although most people with COVID‐19 recover fully within 1 month after infection, approximately 6% of cases are reported to develop serious long‐term sequelae post‐infection [3]. Long COVID, post‐COVID‐19 condition (PCC), post‐acute sequelae of SARS‐CoV‐2 (PASC) and long‐haul COVID are interchangeably used to describe the persistent and chronic symptoms following acute infection [3, 4]. The World Health Organization (WHO) defined PCC as occurring in patients with a history of probable or confirmed SARS‐CoV‐2 infection, usually three months from the acute infection, with symptoms that last for two or more months and that an alternative diagnosis cannot explain [3]. Patients with PCC exhibit a constellation of symptoms such as chronic fatigue, shortness of breath, cognitive disturbances, sleep disturbances, dyspnoea, myalgia and post‐exertional malaise, which indicate interconnected consequences of disruptions in multiple body systems [5, 6, 7, 8, 9, 10, 11, 12, 13]. Currently, there is an absence of a universal standard laboratory‐based diagnostic tests, hence diagnosis for PCC is reliant on the fulfilment of clinical case definitions [1, 3, 14].

Although the pathomechanism underlying the extended persistent symptoms exhibited in PCC is yet to be elucidated, research has demonstrated structural and functional dysregulation of neurological, immunological, cardiovascular, pulmonary and metabolic systems [5, 9, 15, 16, 17, 18, 19]. A key feature is immune dysregulation, potentially induced by numerous factors such as the presence of viral reservoirs, viral fragments, metabolic dysregulation and complex immune responses to SARS‐CoV‐2 infection [18, 20, 21]. Immunological dysregulations such as persistent activation of immune cells, such as neutrophils and monocytes, altered immune communication, exhausted T cells, altered B cell function, elevated cytokine levels and functionally impaired natural killer (NK) cells in PCC, have been reported in various studies [13, 18, 19, 22, 23, 24]. Furthermore, previous research in severely ill COVID‐19 patients has reported suppression of genes involved in cytotoxic activity in CD8 T lymphocytes and NK cells, which was associated with disease severity [25].

In humans, NK cells are part of the innate immune system that form the first line of defence against viral infections by both lysing target cells and producing early immunoregulatory cytokines [26]. They are identified by the surface expression of the neural cell‐adhesion molecule CD56 (positive), the low‐affinity Fc IgG receptor III (FcγRIII, CD16) and negative expression of the CD3 T cell marker [27, 28]. Functionally, the CD3^−^CD56^dim^CD16^bright^ subset has cytotoxic properties, and the CD3^−^CD56^bright^CD16^dim^ subset has immunoregulatory roles [26, 29]. Persistent NK cell functional disturbances associated with disease severity were reported in patients with COVID‐19 [30]. The regulation of NK cell function is reliant on a synergistic network of receptors, proteins, cytolytic granules and the dynamic mitochondrial function, which all together influence their target killing potential through precise regulation of intracellular Ca^2+^ ions, that modulates their effector efficacy [27, 29, 31, 32]. In activated NK cells, Ca^2+^ drives signalling, making the ion essential for lytic granule polarisation, immune synapse formation and exocytosis of cytolytic proteins [33].

Ca^2+^ ions serve as highly crucial cellular signalling messengers for various biological processes such as cell growth, metabolism, gene transcription, muscle contraction and cell death [34]. Importantly, maintaining Ca^2+^ homeostasis is crucial. Ca^2+^ permeable ion channels such as transient receptor potential (TRP) channels and calcium‐release‐activated calcium (CRAC) channels, together with a dynamic network of organelles such as the endoplasmic/sarcoplasmic reticulum (ER/SR) and the mitochondria, synergistically function to precisely mediate cytoplasmic Ca^2+^ entry and intracellular signalling processes [34, 35, 36]. The mitochondria play a key role as adenosine triphosphate (ATP) producers; in addition, they are also involved in various interconnected processes, including cell growth, information transmission, immune responses, Ca^2+^ storage and regulation [37, 38]. Intracellular Ca^2+^ entry is facilitated by various mechanisms including in response to receptor stimulation, depletion of Ca^2+^ stores, changes in membrane potential electrical signalling and binding of specific ligands; however, the depletion of intracellular Ca^2+^ stores induces the store operated Ca^2+^ entry (SOCE), a major Ca^2+^ signalling pathway that leads to activation and regulation of various Ca^2+^ sensitive ion channels in immune cells [39, 40]. During SOCE activation, the mitochondrial network regulates Ca^2+^ mobilization by actively taking up Ca^2+^, thereby indirectly inhibiting inactivation of Ca^2+^ permeable ion channels [41]. The process prolongs cytosolic Ca^2+^ influx, which is required for immune cell activation [32, 39, 41].

Importantly, since NK cells require Ca^2+^ to exert their target effector cytotoxicity [42, 43, 44], in mylagic encephalomyelitis or chronic fatigue syndrome (ME/CFS), reduced NK cell cytotoxicity was linked to decreased Ca^2+^ influx via the transient receptor potential melastatin 3 (TRPM3) ion channel in previous studies [45, 46, 47]. ME/CFS is a complex multisystemic illness, and research over the past few years has reported a considerable overlap in clinical manifestations and pathomechanisms with PCC [48, 49, 50]. In addition, it is hypothesised that SARS‐CoV‐2 is a potential infectious trigger for ME/CFS [51, 52, 53], and more recently, electrophysiology investigations reported impairment of the TRPM3 ion channel in NK cells from patients with ME/CFS and PCC; however, further research is required [54].

TRPM3 belongs to a family of transient receptor potential (TRP) ion channels, a large and diverse family of nonselective cation channels that respond to a wide range of chemical and physical stimuli [55]. TRPM3 is a Ca^2+^ permeable polymodal ion channel that is expressed in a wide variety of tissues and cells, but is highly expressed in the brain, spinal cord, eye, pituitary gland, kidney, adipose tissue and pancreatic beta cells [56, 57, 58]. TRP channels are involved in numerous critical physiological processes, including temperature sensing, taste, vision, nociception, epithelial ion transport, and mineral homeostasis [56, 57, 58]. Therefore, due to their biological roles and wide expression in the body, dysregulation of expression or activity of TRP channels has been linked to some disease onset, such as neuropathic pain, neurodegenerative diseases, and cancer, collectively referred to as TRP‐related channelopathies [59, 60, 61]. TRPM3 ion channel impairment has also been linked to various illnesses such as intellectual disability, epilepsy, musculoskeletal anomalies, altered pain perception, glaucoma and ME/CFS [62, 63, 64].

The TRPM3 ion channel can be activated by both thermal and chemical stimuli, such as the endogenous neurosteroid pregnenolone sulfate (PregS), and reversibly inhibited by ononetin [65, 66]. Furthermore, the TRPM3 ion channel was indicated to form plasmalemmal Ca^2+^ channels with Orai1 and stromal interaction molecule 1 (Stim1) in white matter (WM) glia, contributing to increased Ca^2+^ influx in the central nervous system (CNS) cells [67, 68]. The TRPM3 ion channel senses the changes in Ca^2+^ concentration via calmodulin (CaM), a Ca^2+^ binding protein located on the N‐terminus of the channel, leading to either up‐ or downregulation of the channel's activity [69, 70]. Activation of the TRPM3 ion channel leads to a Ca^2+^ increase, engaging downstream transcriptional regulators such as nuclear factor of activated T cells (NFAT), nuclear factor kappa‐light‐chain‐enhancer of activated B cells (NF‐κB) and c‐Jun N‐terminal kinase (JNK) [71]. This coupling of membrane‐proximal ion flux to transcriptional activity underpins the role of TRPM3 in regulating Ca^2+^‐dependent immune gene expression and downstream immune responses [57, 71, 72].

Given previous research reporting significant dysfunction of TRPM3 and calcium influx in NK cells of PCC patients compared with HCs, the aim of this current investigation was to determine TRPM3 ion channel function on Ca^2+^ influx into the cytoplasm and mitochondria in NK cells using fluorescence live cell imaging from PCC patients compared with HCs.

## 2 Materials and Methods

### 2.1 Study Participants

This research was conducted in accordance with Griffith University's Human Research Ethics guidelines, the National Statement on Ethical Conduct in Human Research (2007, updated 2018), the Australian Code for the Responsible Conduct of Research (2018) and in accordance with the Declaration of Helsinki. The human research ethics application was approved by the Griffith University Human Research Ethics Committee (GU HREC 2022/666), and all participants provided informed and written consent before blood collection. PCC patients and HC were recruited by clinical referral or the National Centre for Neuroimmunology and Emerging Diseases (NCNED) patient database. Between May 2024 and May 2025, eight PCC patients and eight age and sex matched HC volunteered for this study. Patients with PCC were required to have received a clinical diagnosis and meet the clinical case definition by Delphi consensus published by the WHO [3], while HC participants were in good health without incidence of fatigue or evidence of illness. Participants were excluded from this study if pregnant, breastfeeding, reported previous alcohol abuse, obese (body mass index (BMI) ≥ 30), aged under 18 years or over 65 years, or suffered from chronic illness such as autoimmune diseases, cardiovascular disease, diabetes, metabolic syndrome, thyroid disease, malignancies, insomnia, chronic fatigue, or primary psychological disorders. Participants were excluded if they were routinely taking any pharmacological agents that directly or indirectly influence TRPM3 or Ca^2+^ signalling. Participants were provided with the option to cease any conflicting medications for a minimum of 14 days before blood donation after approval from their physician.

All participants completed an online questionnaire providing sociodemographic background, medical history, medications taken, and quality of life (QoL) assessed using the 36‐item short form health survey (SF‐36) and the WHO Disability Assessment Schedule (DAS). The SF‐36 was scored across eight domains, including physical functioning, role limitations due to physical health problems, bodily pain, general health perceptions, energy/vitality, social functioning, role limitations due to personal or emotional problems, and emotional wellbeing/general mental health. Domains were scored on a scale of 0% to 100%, whereby 0 indicated very severe, and 100 indicated no symptom [73]. The WHO DAS assessed functional capacity across six domains of life: cognition, mobility, self‐care, getting along, work and school participation, general life activities, and participation in society. Each of the 36 items of the WHO DAS 2.0 was scored on a five‐point scale: none, mild, moderate, severe, and extreme or cannot do. The subscale scores were generated by first converting each item score into the corresponding, predefined weighted values as outlined in the WHO DAS 2.0 manual (available online at: https://apps.who.int/iris/handle/10665/43974.) [74]. Low percentage scores were associated with no disability, while a high degree of difficulty or greater disability was represented by higher percentages (0 = no symptom, 100 = very severe) [75]. Participants with PCC reported on the presence, frequency, and severity of symptoms. Symptoms were categorised in 10 diverse groups, including cognitive difficulties, pain, sleep disturbances, cardiovascular, gastrointestinal, respiratory, urinary, immunological, and thermoregulatory intolerances, and sensory disturbances.

### 2.2 Natural Killer Cells Isolation

84 mL of whole blood was collected in ethylenediaminetetraacetic acid ([EDTA], Becton Dickinson, BD Biosciences, San Diego, CA, USA) tubes from the antecubital vein of each participant via venepuncture. All blood collections were performed between 6:30 am and 10:00 am at convenient locations for participants, including the Gold Coast campus of Griffith University, Robina Hospital, patient homes, or private laboratories in South East Queensland and North‐East New South Wales. Full blood count was performed within 4 h of collection for each participant, analysing red blood cell count, white blood cell count, and granulocyte cell count at Gold Coast University Hospital or private laboratories in Australia that are NATA certified.

All samples were de‐identified using a unique alphanumeric code before arriving at the NCNED laboratories. PBMCs were isolated from 80 mL of whole blood samples by density gradient centrifugation (Ficoll Paque Plus (Cytiva), GE Healthcare, Uppsala, Sweden), as previously described [76, 77]. Cell counts and cell viability were determined using trypan blue stain (Invitrogen, Carlsbad, CA, USA) and an automatic cell counter (TC20 Automated cell counter, Bio‐Rad Laboratories, Hercules, CA, USA).

NK cells were isolated from PBMCs by immunomagnetic selection using the EasySep human NK cell enrichment kit (Stem Cell Technologies, Vancouver, BC, Canada). The purity of isolated NK cells was determined by CD3^−^CD56^+^ phenotypic surface expression using flow cytometry. NK cells were incubated for 20 min at room temperature in the presence of CD3 PE‐Cy7 (5 µL/test) and CD56 APC (20 µL/test) monoclonal antibodies (BD, Biosciences). Cells were acquired at 10,000 events using the BD X20 flow cytometer (BD Biosciences). Acceptable NK cell purity was ≥75% (Figure S1).

### 2.3 Cell Staining

Cells were initially prepared and washed in 0 mM Ca^2+^ EDTA‐free buffer, unless otherwise stated. The 0 mM Ca^2+^ buffer was prepared in milliQ water and contained Sodium chloride (NaCl) 140 mM, Potassium chloride (KCl) 5.4 mM, Magnesium chloride (MgCl) 2 1.0 mM, and HEPES 10 mM. The pH was adjusted to 7.40 ± 0.05 using NaOH, and the osmolality was adjusted to 300 ± 10 mOsm/L using D‐glucose.

NK cells were adhered to 24‐well glass bottom plates (Celvis, P24‐1.5H‐N) using Corning Cell‐Tak cell and tissue adhesive (Bio‐Strategy, Campbellfield, Vic, Australia) as previously described [78]. For specific mitochondrial identification and Ca^2+^ indication, NK cells were stained with 1 µM Mito Tracker Green FM for 30 min at 37°C, washed, then stained with 1 µM Hoechst 33342 and 1 µM Rhod‐2 AM in the presence of 0.02 % Pluronic F127 (Thermofisher, Waltham, MA, USA) for 15 min at room temperature (RT). To promote sequestration of Rhod‐2 AM into the mitochondria, Rhod‐2 AM was first reduced to a colourless nonfluorescent dihydrorhod‐2 AM by adding a small solid excess of sodium borohydride (NaBH_4_). For cytosolic calcium determination, NK cells were stained with 1 µM Fluo‐8‐acetoxymethyl ester (AM) (Abcam, Cambridge, UK) in the presence of 0.02% Pluronic F127 for 30 min at 37°C. After a wash cycle, cells were pre‐depleted with 1 µM Thapsigargin (Sigma‐Aldrich) for 15 min at RT, then left for a further 15 min, allowing de‐esterification of Fuo‐8‐AM and Rhod 2‐AM, ready for imaging.

### 2.4 Live‐Cell Imaging

To investigate TRPM3‐dependent intracellular Ca^2+^ mobilization, pharmacomodulations were administered via a continuous gravity perfusion system in 1.8 mM Ca^2+^ buffer. The 1.8 mM Ca^2+^ buffer was prepared in milliQ water and contained NaCl 140 mM, KCl 5.4 mM, Calcium chloride (CaCl_2_) 1.8 mM, MgCl_2_ 1.0 mM, and HEPES 10 mM. The pH was adjusted to 7.40 ± 0.05 using NaOH, and the osmolality was adjusted to 300 ± 10 mOsm/L using d‐glucose. Stained NK cells adhered to 24‐well glass‐bottom plates were imaged using the Evident IXplore IX83 SpinSR Super Resolution microscope system (60× objective, oil immersion, spinning disk confocal configuration) with a Hamamatsu ORCA‐Fusion camera and Olympus Cellsens Dimension 4.2 acquisition software (Evident Corporation, Tokyo, Japan). Fluo‐8 fluorescence was acquired at excitation/emission (Ex/Em) 489/508 nm, Mito Tracker Green FM fluorescence at Ex/Em: 490/516 nm, Rhod‐2 fluorescence at 552/581 nm and Hoechst 33342 at Ex/Em: 341/542 nm wavelengths. Time‐lapse images were acquired at 15 s intervals. All experiments were performed at RT (20–25°C). For TRPM3 stimulation, three wells were recorded per participant (Fluo‐8 alone well, Fluo‐8 combined with Rhod‐2 well, Mitotracker Green + Rhod‐2 + Hoechst 33342), the baseline was recorded in 0 mM Ca^2+^ for 60 s, sequentially followed by addition (for 3 min each) of 1.8 mM Ca^2+^, then activation with 50 µM PregS (in vitro technology), ending the experiment by addition of 1 µM Carbonyl cyanide 4‐(trifluoromethoxy)phenylhydrazone (FCCP) to monitor and indicate mitochondrial uncoupling. The perfusion line was adequately washed with 0 mM Ca^2+^ solution between subsequent recordings. To confirm Ca^2+^ influx via TRPM3 ion channel mediation, Ononetin was used for TRPM3 inhibition, and the baseline was recorded in 0 mM Ca^2+^ for 60 s, sequentially followed by addition (all for 3 min each) of 1.8 mM Ca^2+^, inhibition by Ononetin alone, combined modulation using PregS and Ononetin, TRPM3 activation using PregS alone and ending the experiment with mitochondrial uncoupling using 1 µM FCCP (Figure S3).

### 2.5 Image Analysis

**FIGURE 1:**
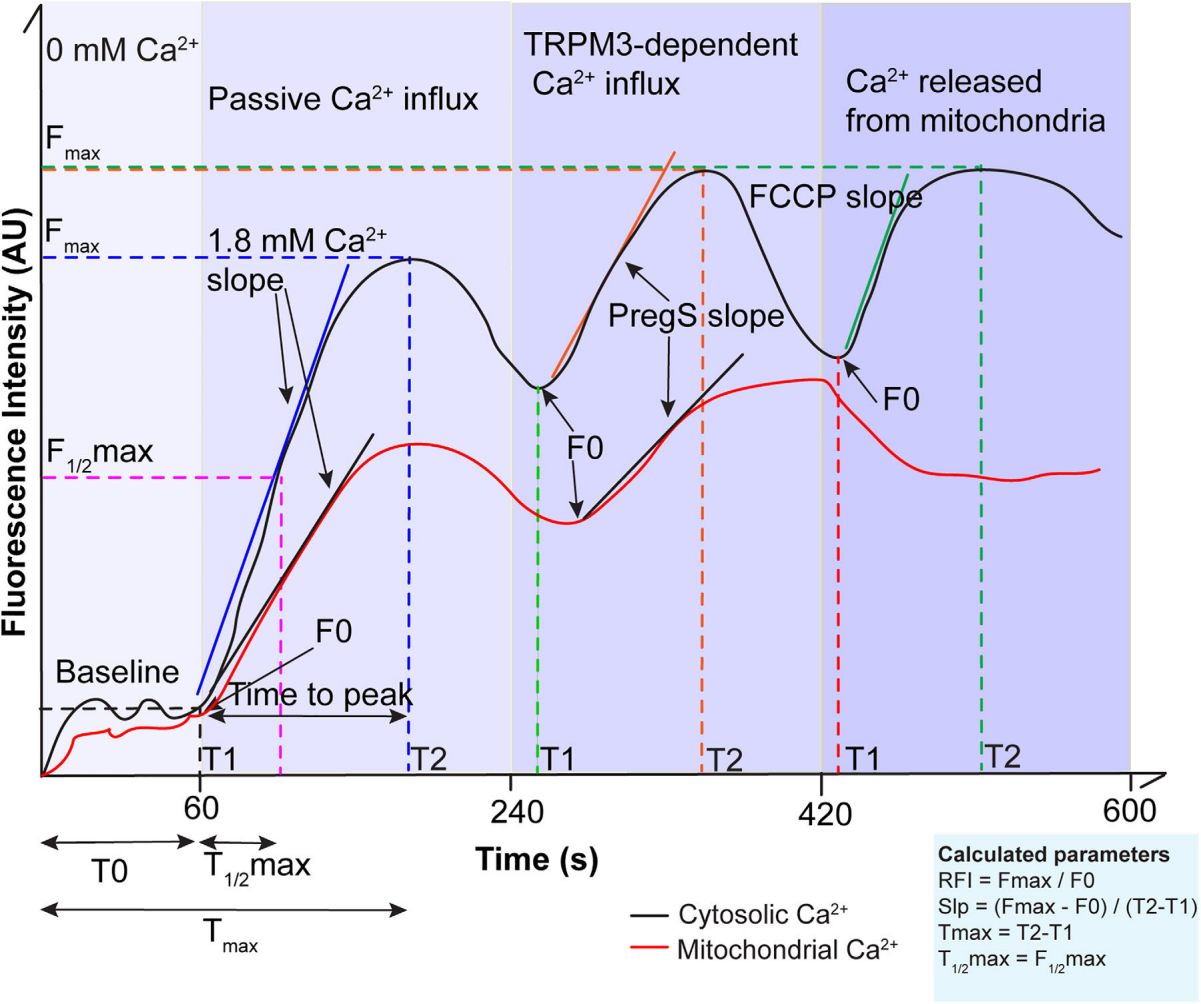
Illustration of analysis of cytosolic and mitochondrial Ca^2+^ influx in NK cells. Measurements were performed during different conditions. Baseline fluorescence was first measured at resting conditions for 60 s, followed by measurement of passive Ca^2+^ influx for 3 min, TRPM3‐dependent Ca^2+^ influx for 3 min, and Ca^2+^ release from the mitochondria following FCCP treatment for another 3 min. The dotted lines indicate F0s and corresponding *T*_1_s for each stimulation phase (black, baseline; magenta, *F*_1/2_max; blue, 1.8 mM Ca^2+^response; orange, PregS response; and green, FCCP response. Fluorescence intensity raw data output was presented in Excel spreadsheet format, then copied and analysed using programmable spreadsheets as illustrated in Figure 1. Relative fluorescence = *F*_max_/*F*_0_; slope = [(*F*_max−_ *F*_0_)/(*T*_2_−*T*_1_)]; half‐time (*T*_1/2_) of Ca^2+^ influx = *T*_1/2_max]. AU, arbitrary units; Ca^2+^, calcium; *F*_0_, minimum fluorescence: *T*_0_: time at baseline; *F*_max_: fluorescence at maximum peak; *T*_1_: Time at minimum fluorescence intensity; *T*_2_: Time at maximum fluorescence intensity; *F*_max_, maximum fluorescence; *F*_0_, minimum fluorescence; PregS, pregnenolone sulfate; FCCP, carbonyl cyanide 4‐(trifluoromethoxy) phenylhydrazone; RFI, relative fluorescence intensity; Slp, slope.

Time‐lapse image data were extracted using Olympus cellSens Dimension Desktop 4.3 software by selecting regions of interest (ROIs) per well. The background was subtracted by drawing an ROI on an area without visible cells. Cells were selected based on good morphology (cells with intact membranes) and their presence throughout the duration of the recording. ROIs around Mito Tracker Green FM perimeter and Rhod‐2 colocalization were used to identify the mitochondrial network. For additional insights, colocalization of the two stains was measured using the ImageJ [JACop (Just Another Colocalization Plugin) software [79], using Pearson's correlation coefficient (Pearson's *r*) and Manders’ Colocalization Coefficients (MCC). Pearson's correlation coefficient was calculated on a pixel‐by‐pixel basis to assess the linear correlation between fluorescence intensity distributions in the two channels, yielding values ranging from −1 to +1, where higher positive values indicate stronger covariance of signal intensities. Manders’ coefficients (M1 and M2) were computed following intensity thresholding to determine the proportion of one fluorophore's signal overlapping with the other, independent of signal proportionality, thereby providing a measure of spatial co‐occurrence. M1 = red, fraction of Rhod‐2 overlapping green (Mitotracker Green); M2 = fraction of Mitotracker Green overlapping red (Rhod‐2). Fluorescence intensity raw data output was presented in excel spreadsheet format, then copied and analysed using programmable spreadsheets as illustrated in Figure 1 [baseline fluorescence: recorded from 0–60 s in 0 mM Ca^2+^ solution; F_0_: minimum fluorescence; F_max_: fluorescence at maximum peak; *T*_1_: time at minimum fluorescence intensity; T_2_: time at maximum fluorescence intensity; relative fluorescence = *F*_max_/*F*_0_; slope = [(*F*_max−_ *F*_0_)/(*T*_2_−*T*_1_)]; half‐time (*T*_1/2_) of Ca^2+^ influx = *T*_1/2_max].

### 2.6 Biological and Chemical Reagents

EDTA Tubes (product code: 367839) for blood collection were purchased from BD Biosciences. Ficoll paque plus (product code: GEHE17‐1440‐03), used for PMBCs isolation and Cell‐Tak cell adhesion (product code: BDAA354240, Bio‐Strategy), was purchased from Bio‐Strategy, Australia. EasySep human NK cell enrichment kits (product code: 19055) and EasySep buffer (product code: 20144) were purchased from Stem Cell Technologies. The following reagents were purchased from Thermo Fisher Scientific: RPMI 1640 (product code: 11835030), HEPES, Mito Tracker TM Green FM (M7514), Rhod 2 AM (R1245MP), Hoechst 33342 (H3570) and Pluronic F‐127 (P3000MP). A 24‐well glass‐bottom plate with high‐performance #1.5 cover glass (product code: P24‐1.5H‐N) was purchased from Cellvis, USA. NaHCO_3_ (product: S5761‐500g), NaCl (product code: 793566‐500G), KCl (product code: 793590‐500G), CaCl_2_ (product code: C5670‐100G), MgCl_2_ (product code: 63069–100ML), NaBH_4_ (product code: 452882‐100g), FCCP (product code: C2920), Thapsigargin (product code: T9033‐1MG), DMSO (product code: D2650‐5×10 mL) were purchased from Sigma‐Aldrich. PregS (product code: RDS537650) and Ononetin (product code: RDS514350) were purchased from In Vitro Technologies. Stock solutions for PregS, Ononetin, Thapsigargin, Fluo‐8 AM, Mitotracker Green, and Rhod‐2 AM were prepared in dimethylsulfoxide (DMSO) (product code: D2650, Sigma‐Aldrich).

### 2.7 Statistical Analysis

Participant demographics data were analysed using Statistical Package for the Social Sciences (SPSS) v26 (IBM Corp, Version 29, Armonk, NY, USA). Flow cytometry data were exported from the BD X20 flow cytometer and analysed for purity using GraphPad Prism, version 9 (GraphPad software Inc., CA, USA). Extracted time‐lapse fluorescence intensity imaging data were analysed using a programmable Excel sheet, Origin software version 2024b (OriginLab Corporation, Massachusetts, USA) for quality analysis check, and GraphPad Prism for statistical analyses. The robust regression and outlier removal (ROUT) method was used to identify and remove outliers from the analysis. For statistical comparison, the normality of distribution of parameters was assessed using the Shapiro–Wilk test. The Mann–Whitney *U* test was performed on nested nonparametric data. The data are presented as median (Mdn) values and mean ± standard deviation (SD) for descriptive statistics. Significance was set at *p* < 0.05.

## 3 Results

### 3.1 Participants Demographics

Sixteen participants were included in this study: *N* = 8 HC and *N* = 8 PCC patients who met the WHO clinical case definition [3] and did not report other fatigue‐related illnesses that explained their symptoms. Demographic data are presented in Table 1. For PCC patients, the average age and SD were 42.50 ± 15.57 years and 45.00 ± 14.83 years for HC. Three HC and three PCC were female participants. Five HC and five PCC were male participants. There was no significant difference in gender, age, and BMI among groups.

**TABLE 1:** Variations in QoL and disability scores between HC and PCC patients, based on WHO DAS and SF‐36 surveys.

		HC	PCC	*p*‐value
Age (years)		45.00 ± 14.83	42.50 ± 15.57	0.951
Gender	Male	5 (62.5)	5 (62.5)	
	Female	3 (37.5)	3 (37.5)	
BMI (kg/m^2^)		25.12 ± 5.29	24.38 ± 3.73	0.957
SF‐36 (%)				
Physical functioning		98.57 ± 2.44	49.36 ± 26.25	**0.001**
Physical role		100.00 ± 0.00	16.41 ± 25.65	**0.001**
Pain		94.29 ± 5.35	44.38 ± 33.29	**0.001**
General health		73.81 ± 20.37	34.38 ± 17.92	**0.001**
Social functioning		92.86 ± 18.90	25.00 ± 21.13	**0.001**
Emotional role		100.00 ± 0.00	67.71 ± 37.12	**0.008**
Emotional wellbeing		80.71 ± 9.32	53.75 ± 27.22	**0.004**
Vitality		70.54 ± 8.63	21.09 ± 19.75	**0.001**
WHODAS (%)				
Communication and understanding		6.77 ± 9.94	34.90 ± 31.34	**0.022**
Mobility		3.13 ± 8.84	36.88 ± 27.25	**0.004**
Self‐care		0.78 ± 2.21	20.31 ± 26.46	**0.035**
Interpersonal relationships		7.03 ± 7.04	35.94 ± 34.19	**0.023**
Life activities		3.13 ± 8.84	51.56 ± 33.53	**0.001**
Work/school participation		10.94 ± 12.83	37.50 ± 40.78	**0.132**
Participation in society		5.04 ± 6.58	35.98 ± 28.68	**0.008**

*Note*: High SF‐36 scores reflected good QoL, and low scores suggested poor QoL (0 = severe symptoms and 100 = no symptoms). Low WHO DAS scores were associated with no functional limitations, and high scores reflected severe functional impairments (0 = no symptom, 100 = very severe). Data is presented as mean ± SD or *N* (%). Values of *p* < 0.05 are bolded.

Abbreviations: BMI, body mass index; HC, healthy controls; PCC, post‐COVID‐19 condition; SF‐36, 36‐item short‐form health survey; WHODAS, World Health Organization disability assessment schedule.

WHO DAS and SF‐36 surveys were used to assess the QoL and disability for all participants. Data are shown in Table 1. There was a significant difference identified between HC and PCC patients in all domains of SF‐36, including physical functioning, physical role, social functioning and vitality (*p* = 0.001), general health (*p* = 0.001), pain (*p* = 0.001), emotional wellbeing (*p* = 0.004) and emotional role (*p* = 0.008). WHO DAS scores were significantly different between the two groups in all domains except work/school participation (*p* = 0.132). Communication and understanding (*p* = 0.022), mobility (*p* = 0.004), life activities (*p* = 0.001), self‐care (*p* = 0.035), interpersonal relationships (*p* = 0.023) and participation in society (*p* = 0.008).

#### 3.1.1 Participant Demographics Quality of Life and Disability Scores (SF‐36 and WHODAS)

#### 3.1.2 Symptoms and Onset Details From Post‐COVID‐19 Group

The average age and SD of illness onset for PCC patients were 40.13 ± 15.45 years, with 2.68 ± 0.51 years as the average and SD for disease duration. All 8 (100.0%) PCC patients reported experiencing fatigue and sleep disturbances, while 7 (87.5%) reported experiencing cognitive difficulties and pain. Sensory, immune, gastrointestinal, and cardiovascular disturbances were declared by 6 (75.0%), respiratory problems were reported by 4 (50.0%) and thermostatic intolerance by 5 (62.5%) of PCC patients. Only 1(12.5%) PCC patient reported experiencing urinary disturbances. All symptoms and frequency data are detailed in Table 2.

**TABLE 2:** The symptoms and onset details from the PCC group.

		PCC
Age of onset (years [mean ± SD])		40.13 ± 15.45
Disease duration (years [mean ±SD])		2.68 ± 0.51
Fatigue	Yes No	8 (100.0%) 0 (0.0%)
Cognitive difficulties	Yes No	7 (87.5%) 1 (12.5%)
Pain	Yes No	7 (87.5%) 1 (12.5%)
Sleep disturbances	Yes No	8 (100.00%) 0 (0.0%)
Sensory disturbances	Yes No	6 (75.0%) 2 (25.0%)
Immune disturbances	Yes No	6 (75.0%) 2 (25.0%)
Gastrointestinal disturbances	Yes No	6 (75.0%) 2 (25.0%)
Cardiovascular disturbances	Yes No	6 (75.0%) 2 (25.0%)
Respiratory	Yes No	4 (50.0%) 4 (50.0%)
Thermostatic intolerance	Yes No	5 (62.5%) 3 (37.5%)
Urinary disturbances	Yes No	1 (12.5%) 7 (87.5%)

*Note*: Data presented as mean ± SD or *N* (%).

Abbreviations: *N*, number of participants; PCC, post‐COVID‐19 condition; SD, standard deviation.

#### 3.1.3 Full Blood Count Parameters

A full blood count was performed for all participants. The results were within the normal range for all cases, as defined by pathology service providers that were a NATA‐accredited laboratory. However, PCC patients had significantly elevated haematocrit (*p* = 0.027) and haemoglobin (*p* = 0.031) levels. We concluded this could have been due to dehydration in some of the participants. Full blood count results are shown in Table 3.

**TABLE 3:** Full blood count parameters.

		HC	PCC	*p*‐value
Full blood count	White Cell Count (4.0–11.0 × 10^9^/L)	5.69 ± 1.15	5.81 ± 1.26	0.844
	Lymphocytes (1.0–4.0 × 10^9^/L)	1.75 ± 0.52	1.67 ± 0.34	0.754
	Neutrophils (2.0–8.0 × 10^9^/L)	3.15 ± 0.54	3.42 ± 1.04	0.527
	Monocytes (0.1–1.0 × 10^9^/L)	0.51 ± 0.21	0.45 ± 0.15	0.556
	Eosinophils (< 0.6 × 10^9^/L)	0.24 ± 0.17	0.23 ± 0.08	0.954
	Basophils (< 0.2 × 10^9^/L)	0.05 ± 0.02	0.03 ± 0.02	0.319
	Platelets (140–400 × 10^9^/L)	244.00 ± 29.77	253.38 ± 56.51	0.674
	Red Cell Count (3.8–5.2 × 10^12^/L)	4.79 ± 0.44	5.14 ± 0.44	0.086
	Haematocrit (0.33–0.47)	0.42 ± 0.03	0.45 ± 0.03	**0.027**
	Haemoglobin (115–160 g/L)	140.13 ± 12.52	150.00 ± 10.47	**0.031**

*Note*: Data are presented as mean ± SD. Reference ranges for full blood count parameters have been included in the table.

Abbreviations: FBC, full blood count; HC, healthy controls; *N*, number of participants; PCC, post‐COVID‐19 condition.

### 3.2 Changes in Cytosolic Calcium Influx in Response to PregS Stimulation in NK Cells in Post‐COVID‐19 Condition Patients

**FIGURE 2:**
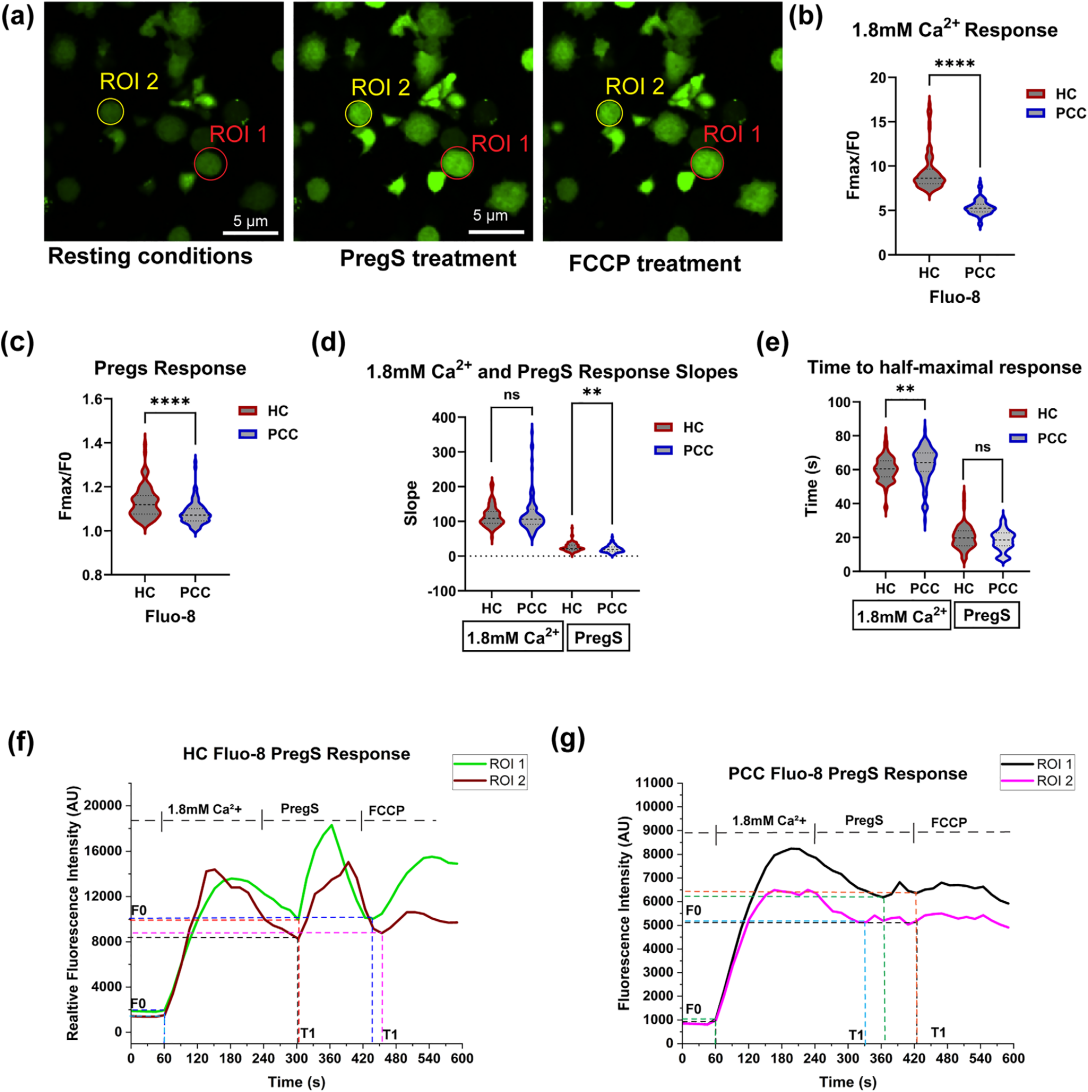
Cytosolic Ca^2+^ influx response in freshly isolated NK cells. (a) Examples of NK cells from a PCC patient stained with Fluo‐8 AM intracellular calcium indicator imaged at 60×, at the start of the experiment and after stimulation with PregS and FCCP, respectively. Group comparisons for pooled data (b–e) were performed with unadjusted two‐sided Mann–Whitney *U* tests on nested data. The violin plots show median (central line) and quartile levels for pooled data. *Represents significant differences after group comparison. (b) Comparison of pooled cytosolic Ca^2+^ influx in PCC (*N* = 8, *n* = 552 NK cells) and HC (*N* = 8, *n* = 540 NK cells) following addition of 1.8 mM Ca^2+^ to NK cells. Fluorescence intensity is shown as the ratio of measured intensity (Fmax) and the minimum fluorescence value (F0). (c) Response to 50 µM PregS stimulation in HC (*N* = 8, *n* = 415 NK cells) and PPC (*N* = 8, *n* = 393 NK cells), (d) the rate (slope) of response to 1.8 mM Ca^2+^ and PregS. (e) *T*_1/2_ response for 1.8 mM Ca^2+^ and PregS. (f) Example of Fluo‐8 AM time lapse fluorescence intensity curves for NK cells from an HC, (g) time lapse fluorescence intensity curves for ROI 1 and ROI 2 shown in (a) from a PCC patient. The dotted lines indicate *F*_0_s and corresponding *T*_1_s for each stimulation condition (f: Light blue, baseline; black and orange, PregS responses; blue and magenta, FCCP responses; g: green, baseline; light blue and green, PregS response and black and orange, FCCP responses). Ca^2+^, calcium; HC, healthy control; PCC, post‐COVID‐19 condition; ROI, region of interest; FCCP, carbonyl cyanide 4‐(trifluoromethoxy) phenylhydrazone; *F*_max_, maximum fluorescence; *F*_0_, minimum fluorescence; PregS, pregnenolone sulfate. Imaging analysis was performed using cellSens Dimension Desktop by drawing perimeters around ROIs, then edited using Adobe Illustrator. Time‐lapse fluorescence Graphs were constructed using Origin software.

Fluorescence live cell imaging measured the relative amount of Ca^2+^ influx and rate of response using Fluo‐8 AM Ca^2+^ indicator, as illustrated in Figure 1. Results are presented in Figure 2. Cytosolic Ca^2+^ influx data are presented as the relative change in fluorescence intensity (Fmax/F0) of Fluo‐8 AM, as shown in Figure 2a. The average Ca^2+^ influx amplitude into the cytosol was significantly reduced in NK cells from PCC patients (Mdn = 5.26, 3.31–8.16) compared with HC (Mdn = 8.62 (7.11–17.0) (Figure 2b, *p* < 0.0001) after depletion of Ca^2+^ stores using 1 µM thapsigargin, followed by addition of 1.8 mM Ca^2+^ buffer. Although there was no significant difference in the rate of response (slope, *p* = 0.2209, Figure 2d) to 1.8 mM Ca^2+^ buffer, the time to half‐maximal (*T*_1/2_) response was significantly longer in PCC patients compared with HC (Figure 2e, *p* = 0.0016). Stimulation of the TRPM3 ion channel with 50 µM PregS successfully induced a significantly higher rate of response (Figure 2d, slope, *p* < 0.0001) in HC compared with PCC patients. In contrast, following PregS stimulation, the slope (Figure 2d) and cytosolic Ca^2+^ influx amplitude (Figure 2c, *p* < 0.0001) were significantly reduced in PCC (Mdn = 1.06, 1.00–1.19) compared with HC (Mdn = 1.11, 1.01–1.39), indicating altered channel function. The *T*_1/2_ response to PregS stimulation was comparable between groups (Figure 2e, *p* = 0.1017). Representative time‐lapse fluorescence intensity responses of NK cells in HC are shown in Figure 2f,g, respectively.

### 3.3 Changes in Calcium Mobilization in the Mitochondria in Response to PregS Stimulation in NK Cells in Post‐COVID‐19 Patients

**FIGURE 3:**
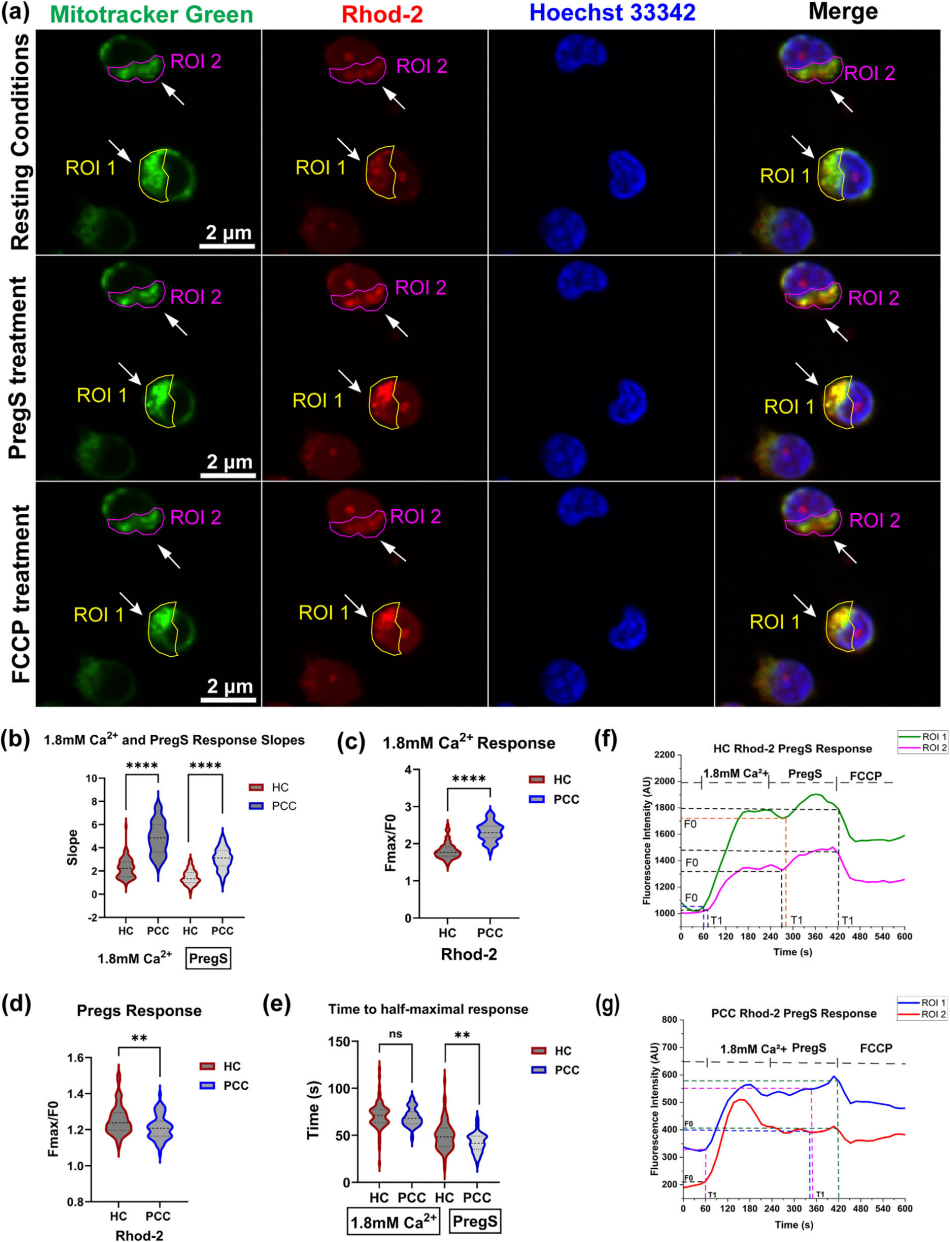
Fluorescence imaging of calcium influx into the mitochondria in NK cells. (a) Representative images of NK cells from an HC participant stained with Mitotracker Green FM (green), Rhod‐2 AM (red), and Hoechst 33342 (blue) taken at the start of the experiment, after PregS and FCCP stimulation, respectively, at 60×. Arrows indicate ROI perimeter around mitochondrial network, indicating change in Rhod‐2 fluorescence intensity overtime (increased yellow spots overtime). Group comparisons for pooled nested data (b–e) were performed with unadjusted two‐sided Mann–Whitney *U* tests. The violin plots show median (central line) and quartile levels for pooled data. *Represents significant differences after group comparison. (b) Comparison of pooled rate (slope) of responses to 1.8 mM Ca^2+^ and PregS, in HC and PCC, respectively, (c) 1.8 mM Ca^2+^ influx amplitude in HC (*N* = 8, *n* = 336 NK cells) and PCC (*N* = 8, *n* = 290 NK cells), and (d) PregS stimulation in HC (*N* = 8, *n* = 336 NK cells) and PCC (*N* = 8, *n* = 289 NK cells), respectively. (e) *T*_1/2_ response to 1.8 mM Ca^2+^ and PregS, respectively. (f) Examples of Rhod‐2 time‐lapse fluorescence intensity curves for NK cells from an HC are shown in (a). The dotted lines indicate *F*_0_s and corresponding *T*_1_s for each stimulation condition (blue, baseline; black and orange, PregS response and black, FCCP response). (g) Examples of Rhod‐2 time‐lapse fluorescence intensity curves for NK cells from a PCC patient (magenta and black, baseline; magenta and blue, PregS response and green, FCCP response). Abbreviations: Ca^2+^, calcium; HC, healthy control; PCC, post‐COVID‐19 condition; ROI, region of interest; 4‐(FCCP, carbonyl cyanide trifluoromethoxy) phenylhydrazone; *F*_max_, maximum fluorescence; F_0_, minimum fluorescence; PregS, Pregnenolone sulate. Imaging analysis was performed using cellSens Dimension Desktop by drawing perimeters around ROIs, then edited using Adobe Illustrator. Time‐lapse fluorescence Graphs were constructed using Origin software.

The downstream effect of TRPM3 ion channel impairment on Ca^2+^ influx into the mitochondria was examined using mitochondrial Ca^2+^ indicator Rhod‐2. Results are illustrated in Figure 3. Mitochondrial Ca^2+^ influx data are expressed as the relative change in fluorescence intensity (Fmax/F0) of Rhod‐2 as presented in Figure 3a. Following depletion of internal Ca^2+^ stores, addition of 1.8 mM Ca^2+^ buffer resulted in a significantly faster rate of response (Figure 3b, slope, *p* < 0.0001) and higher mitochondrial Ca^2+^ influx amplitude (Figure 3c, *p* < 0.0001 in PCC (Mdn = 2.30, 1.76–2.83) compared with HC (Mdn = 1.77, 1.42–2.49). However, the *T*_1/2_ response (Figure 3e, *p* = 0.2845) was comparable. Meanwhile, although the slope for PregS response was significantly higher (Figure 3b, slope, *p* = 0.0001) in PCC, the TRPM3‐dependent Ca^2+^ influx amplitude (Figure 3d, *p* = 0.0036) into the mitochondria was significantly reduced in PCC (Mdn = 1.21, 1.07–140) compared with HC (Mdn = 1.24, 1.11–1.50). The *T*_1/2_ response (Figure 3e, *p* = 0.0042) to PregS stimulation was significantly shorter in PCC patients, indicating altered channel function. Figure 2f,g shows representative time‐lapse fluorescence intensity responses of NK cells from an HC and a PCC, respectively. As a monitor to indicate mitochondrial uncoupling signals, the mitochondrial Ca^2+^ influx response in both groups was significantly attenuated when NK cells were treated with the mitochondrial uncoupling agent FCCP (1 µM). To demonstrate a potential downstream signalling of TRPM3‐dependent Ca^2+^ influx into the mitochondria, NK cells stained with Fluo‐8 and Rhod‐2 were imaged simultaneously (Figure S2). We successfully detected TRPM3‐dependent Ca^2+^ influx response and the downstream signalling into the mitochondria in HC, whereby the responses in PCC were highly altered. To confirm Ca^2+^ influx via TRPM3 ion channel mediation, ononetin successfully inhibited TRPM3 activity, which was successfully activated again by PregS stimulation (Figure S3). TRPM3 inhibition and activation in NK cells were apparent when cytosolic and mitochondrial Ca^2+^ responses were plotted against each other. The Ca^2+^ responses from single dye wells (Fluo‐8 alone and Rhod‐2 + Mitotracker Green) were consistent with combined dye wells (Fluo‐8 + Rhod‐2) responses.

### 3.4 Colocalization of Mitotracker Green and Rhod‐2 Signals Over Time in NK Cells in Post‐COVID‐19 Patients

**FIGURE 4:**
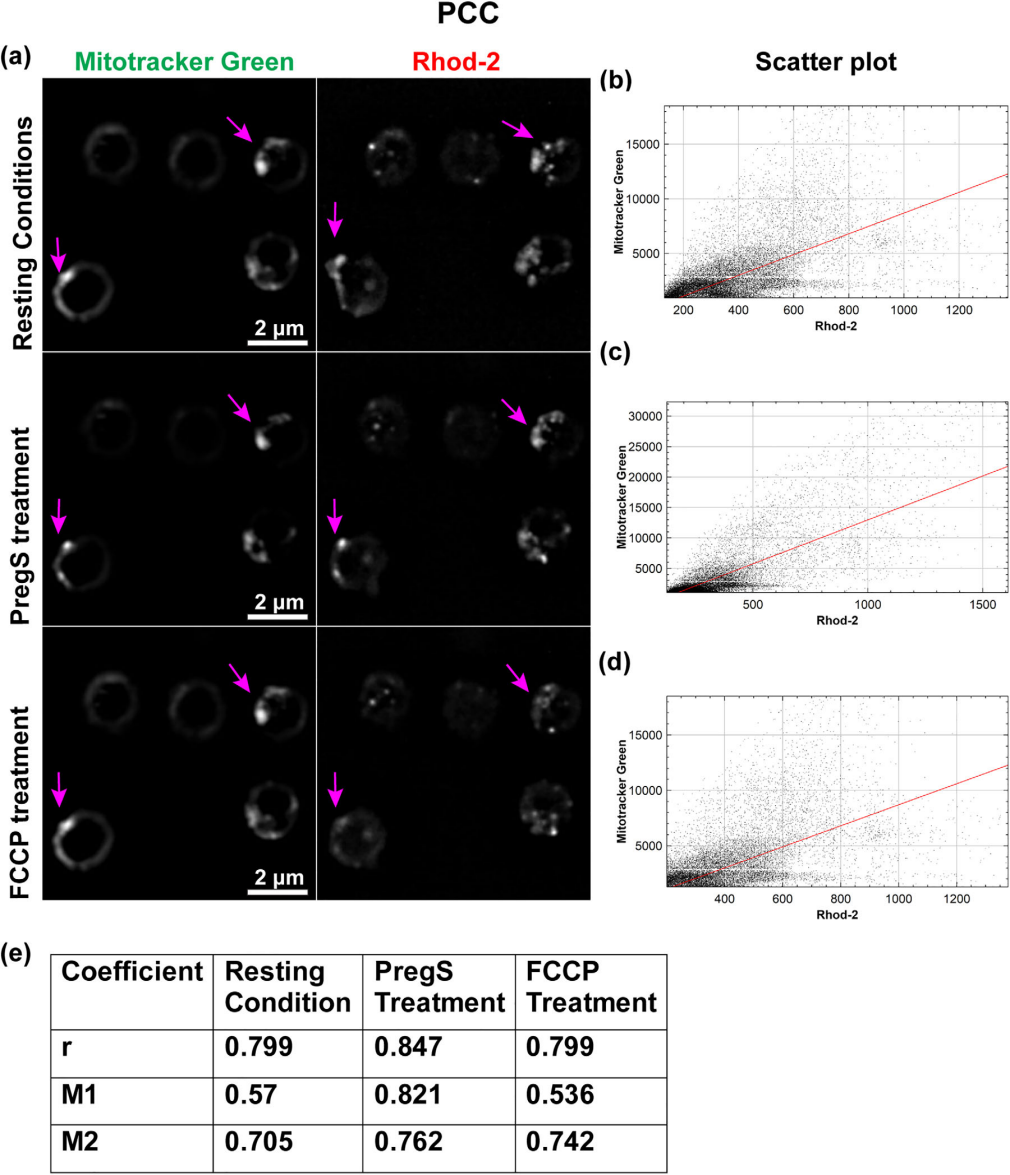
Colocalization analysis with JACop; Pearson and Manders, scatter plots and correlation coefficients. (a) Representative grey scale images of NK cells from a PCC patient stained with Mitotracker Green FM and Rhod‐2 AM exhibiting similar changes in Rhod‐2 fluorescence intensity over time, at 60×, shown in Figure 3. Arrows indicate mitochondrial networks in two cells containing both stains, showing a change in fluorescence intensity overtime. Scatter plots (b–d) correspond to the colocalization of Mitotracker Green FM and Rhod‐2 in response to changes in Ca^2+^ influx in the mitochondria. (b) Corresponding scatterplot of Mitotracker Green and Rhod‐2 fluorescence intensities of NK cells at resting conditions. (c) Corresponding scatterplot of Mitotracker Green and Rhod 2 fluorescence intensities of NK cells after stimulation with PregS, showing increased Rhod‐2 fluorescence intensity, indicating Ca^2+^ mobilization into the mitochondria, consequently, a change in slope by the fitted line and rotation of the dotted cloud toward Mitotracker Green axis. (d) Corresponding scatterplot of Mitotracker Green and Rhod 2 fluorescence intensities of NK cells after treatment with FCCP, showing decreased Rhod‐2 fluorescence intensity as Ca^2+^ efflux from the mitochondria into the cytoplasm, resulting in a change of the slope toward the Rhod‐2 axis and separation of the dotted cloud. (e) Summary of the Pearson's *r* and Manders’ coefficients. Ca^2+^, calcium; Pearson's *r*, Pearson correlation coefficient; PCC, post‐COVID‐19 condition; M1 = Rhod‐2 and M2 = Mitotracker Green, MCC, Manders correlation coefficients; PregS, pregnenolone sulate; 4‐(FCCP, carbonyl cyanide trifluoromethoxy) phenylhydrazone.

For additional insights into a downstream signal into the mitochondria, Ca^2+^ influx into the mitochondria was confirmed using scatterplots demonstrating Mitotracker Green and Rhod‐2 overlay, and changes in Rhod‐2 fluorescence intensity overtime. Pearson's r provided a pixel‐by‐pixel linear correlation between fluorescence intensity distributions in the two channels, while Manders’ coefficients (M1 and M2) provided a measure of spatial co‐occurrence overtime. Changes in the distribution and colocalization of Rhod‐2 with Mitotracker Green in the mitochondria were visible overtime, as illustrated in Figure 4a for PCC and Figure S4 for HC. The degree of colocalization between Rhod‐2 (M1 = red) and Mitotracker Green (M2 = green) over time is presented in Figure 4. The scatterplots (Figure 4b–d) display the intensity range of Mitotracker Green and Rhod‐2. At resting conditions, the distribution of the two stains was positively mapped, with a Pearson's *r* of 0.799, M1 = 0.517, and M2 = 0.705 (Figure 4b). Increased Rhod‐2 fluorescence intensity and overlapping of the two stains were observed following stimulation of NK cells with PregS (Pearson's *r* = 0.847, M1 = 0.821, and M2 = 0.762) (Figure 4c), indicating PregS induced Ca^2+^ influx into the mitochondria. Hence, a change in slope by the fitted line, movement of more dotted points toward the fitted line, and rotation of the dotted cloud toward the Rhod‐2 axis as shown in the scatterplot in Figure 4c. The Ca^2+^ signal of Rhod‐2 was attenuated when NK cells were treated with the mitochondrial uncoupling agent FCCP (1 µM) (Pearson's *r* = 0.799, M1 = 0.536 and M2 = 0.742). Consequently, a change in the slope and separation of the dotted cloud was observed (Figure 4d). A summary of Pearson's *r* indicating strong coordinated signal variation and Manders’ colocalization coefficients showing changes in spatial overlap between fluorescence signals overtime, respectively, is presented in Figure 4e. Together, these complementary metrics enabled a robust quantitative assessment of both signal correlation and subcellular colocalization overtime. Collectively, despite evidence of TRPM3 dysfunction in NK cells from PCC patients, PregS may facilitate compensatory mitochondrial Ca^2+^ uptake, potentially through activation of residual TRPM3 channels or via unknown pathways.

## 4 Discussion

This recent study investigated TRPM3‐dependent Ca^2+^ influx into the mitochondria in NK cells freshly isolated from patients with PCC and HC. We report for the first time the significant decrease in TRPM3‐dependent Ca^2+^ influx into the mitochondria in NK cells from PCC. Results from this study further highlight the role of ion channel dysfunction and consequential decreased Ca^2+^ influx into the mitochondria, suggesting further evidence of TRPM3 dysfunction and Ca^2+^ influx in the pathomechanism of PCC and their adverse impact on cellular organelles, in this instance, the mitochondria. Importantly, these current findings support recent electrophysiological findings of TRPM3 ion channel dysfunction ex vivo in NK cells from PCC patients [54] and further support a common pathomechanism identified in PCC and ME/CFS [45, 46, 47, 54].

Immune dysregulation is reported as a key feature in patients recovering from COVID‐19 disease, with various studies reporting altered immune cell populations, increased presence of inflammatory mediators and reduced cytotoxicity by NK cells [19, 22, 23, 80, 81, 82]. The function and cytotoxicity of NK cells are dependent upon a stable supply of Ca^2+^ ions, with SOCE representing the principal pathway for Ca^2+^ entry [83]. Accordingly, the distribution and functional states of Ca^2+^ permeable ion channels, receptors, pumps and Ca^2+^ storage organelles fundamentally impact Ca^2+^ mobilization across cells [32, 67, 84, 85]. Hence, we initially assessed NK cell responses to the addition of 1.8 mM extracellular Ca^2+^ following depletion of ER Ca^2+^ stores using 1 µM thapsigargin. The addition of 1.8 mM Ca^2+^ led to a slow, sustained, and higher cytosolic Ca^2+^ rise in NK cells from HC compared with PCC, successfully demonstrating a reintroduction of Ca^2+^ to intracellular stores expected in healthy cells. Depletion of intracellular Ca^2+^ stores and inhibition of the sarco/endoplasmic reticulum calcium‐ATPase (SERCA) pump by thapsigargin activate the SOCE and subsequently additional Ca^2+^ permeable channels to replenish the Ca^2+^ stores [86]. Notably, TRP channels, including TRPM3, are proposed to contribute to SOCE, acting as modulators of intracellular Ca^2+^ homeostasis [57, 87]. Thus, a significant reduction in cytosolic Ca^2+^ influx observed in NK cells from individuals with PCC suggests impaired SOCE responses.

The ability of cells to maintain a stable Ca^2+^ homeostasis across cells is vital for activation and initiation of both the innate and adaptive immune response; therefore, the efficiency and speed of Ca^2+^ signalling is crucial for maintaining homeostasis [84]. Over the last few years, various studies have reported persistent immune dysregulation such as compromised B‐cell function, impaired memory T cell responses and reduced NK cell cytotoxicity in PCC [18, 22, 88]. The reduced reintroduction of Ca^2+^ into the cytoplasm of NK cells in PCC after depletion of ER stores found in this study could potentially contribute to previously reported reduced cytotoxicity by NK cells [22, 24, 88, 89]. As Ca^2+^ ions are vital for many vital biological processes, a disturbance to Ca^2+^ signalling could contribute to pathological outcomes involving other body systems, including the immune system, metabolism and neurological dysfunction exhibited in PCC, thus supporting the above claim [90].

Recently, TRPM3 ion channel impairment in NK cells from PCC and ME/CFS was demonstrated using the patch‐clamp technique [54]. TRP channels function as critical sensors of physical and chemical stimuli, regulating inflammatory responses and immune cell function through Ca^2+^‐dependent signalling pathways, thereby shaping and maintaining intracellular and extracellular Ca^2+^ homeostasis [84, 87]. Notably, these channels are expressed in virus‐susceptible host cells, where viral pathogens can alter their expression and activity to exploit TRP‐mediated Ca^2+^ influx for key stages of the viral life cycle, including entry, replication, and egress [91]. This hijacking of host Ca^2+^ signalling machinery frequently results in dysregulated intracellular Ca^2+^ concentrations, contributing to cellular dysfunction [92]. Mutations within TRP channel genes are associated with a spectrum of channelopathies characterised by either gain or loss‐of‐function phenotype, including neurological, metabolic, and immunological disorders [93]. In this context, impaired TRPM3 activity in NK cells from individuals with PCC was first reported using the patch clamp technique, highlighting potential dysfunctional similarities with ME/CFS [54]. Hence, we examined the effect of TRPM3‐dependent Ca^2+^ influx into the cytoplasm. Activation of the TRPM3 ion channel with 50 µM PregS successfully evoked a significantly higher response rate (*p* < 0.0001) in HC, triggering a significantly higher cytosolic Ca^2+^ influx. In contrast, the amplitude (*p* < 0.0001) and rate of Ca^2+^ influx were significantly reduced in PCC compared with HC. These results support previous findings that reported TRPM3 dysfunction in PCC using the electrophysiology technique [54]. The decreased TRPM3 activity observed in NK cells from individuals with PCC may arise from multiple converging mechanisms involving isoform dysregulation and inflammatory modulation. TRPM3 functional integrity is highly dependent on its primary amino acid sequence and structural domains; specifically, alterations within exon 24, which encodes the pore‐forming region of TRPM3, can significantly alter ionic selectivity and reduce Ca^2+^ permeability by modifying electrostatic properties within the channel pore [94]. Additionally, deletions or mutations within exon 13, encoding a critical TRPM3 structural and functional domain known as the region indispensable for channel function (ICF), result in the generation of nonfunctional TRPM3 isoforms [95]. These altered isoforms can exert dominant‐negative effects, interfering with the activity of fully functional channels and thereby attenuating overall TRPM3‐mediated Ca^2+^ influx [95].

TRPM3‐mediated Ca^2+^ mobilization engages broader signalling networks, including phosphorylation and activation of phosphatidylinositol 3‐kinase (PI3K) and mitogen‐activated protein kinases (MAPKs) such as rapidly accelerated fibrosarcoma (Raf), extracellular signal‐related kinases (ERK1/2) and c‐Jun N‐terminal kinase (JNK), which are implicated in the regulation of NK cell activation, degranulation and inflammatory cytokine release [71, 96]. In parallel, ligand binding on natural killer group (NKG) family or natural cytotoxicity receptors (NCR) family receptors evokes Ca^2+^ entry through the SOCE pathway and Ca^2+^‐permeable channels, including TRPM3, activating Ca^2+^‐dependent immune responses [32, 97]. Thus, reduced Ca^2+^ influx rate correlates with lower cell activation and diminished effector functions. Accordingly, impaired TRPM3 function may blunt Ca^2+^ signalling, weaken MAPK/PI3K pathway activation and impair Ca^2+^‐dependent processes required for effective NK cell cytotoxicity. While outside the scope of the current investigation, future research should aim to elucidate the downstream consequences of protein kinase phosphorylation following NK cell activation.

In addition to their role as active Ca^2+^ ion channels, TRP ion channels in the plasma membrane contribute to changes in cytosolic Ca^2+^ by modulating alternative Ca^2+^‐permeable ion channels and driving force pathways, including the SOCE process, voltage‐gated Ca^2+^ channels and changing the membrane potential [85, 98]. Therefore, alteration to their optimal functioning has a potential downstream cumulative effect leading to detrimental cellular consequences, is this instance decrement in Ca^2+^ mobilization in NK cells, potentially contributing to reduced cytotoxicity properties and other disturbances reported in PCC. Further, since TRPM3 is highly expressed in various tissue organs and cells, including the brain, spinal cord, eye, pituitary gland, kidney, adipose tissue and pancreatic beta cells [56, 57, 58], the impaired TRPM3‐dependent Ca^2+^ mobilization may potentially be present in other tissues, contributing to cognitive dysfunction, chronic fatigue and other disturbances exhibited in PCC.

Further, attributable to their dynamic function, the mitochondria have the ability to accumulate and store Ca^2+^ ions, contributing to a stable intracellular homeostasis [35, 99]. Recently, TRPM3 activation was found to trigger mitochondrial Ca^2+^ loading in nociceptive dorsal root ganglion (DRG) neurons, highlighting dynamic mitochondrial activity downstream of TRPM3 activation [100]. Given that these proteins and organelles are functionally coupled, it was deemed relevant to investigate TRPM3‐dependent Ca^2+^ influx into the mitochondria in NK cells from PCC patients. Following depletion of internal Ca^2+^ stores, addition of 1.8 mM Ca^2+^ led to significantly higher Ca^2+^ influx amplitude into the mitochondria, response rate (slope) and *T*_1/2_ response in PCC compared with HC (*p* < 0.0001). Although stimulation of NK cells with PregS pronouncedly activated the TRPM3 ion channel (*p* < 0.0001) in HC, and triggered significant Ca^2+^ influx, Ca^2+^ loading into the mitochondria and *T*_1/2_ response (*p* = 0.0001) was significantly reduced in PCC patients compared with HC. On the other hand, NK cells from PCC had a faster rate of Ca^2+^ influx (slope, *p* = 0.0001) into the mitochondria, and the TRPM3 activation *T*_1/2_ was shorter compared with HC. The rapid and higher Ca^2+^ rise into the mitochondria in NK cells from PCC contrasts with the slower and lower Ca^2+^ rise observed in the cytoplasm. Based on our current results, given that Ca^2+^ influx into the cytoplasm was significantly reduced in NK cells from PCC patients, it is possible that Ca^2+^ storage amount in the mitochondria is reduced, consequently causing rapid (increased slope) Ca^2+^ uptake for a longer time (longer *T*_1/2_ response) to replenish Ca^2+^ stores. Indeed, a compensatory mechanism to rapidly replenish Ca^2+^ stores and buffering by the mitochondria is proposed [90, 101]. In addition, alterations in the Ca^2+^ flux pathways and speed were found to subsequently increase the Ca^2+^ influx speed into the mitochondria and amount via the mitochondrial calcium uniporter (MCU), even at relatively low cytosolic Ca^2+^, potentially leading to mitochondrial Ca^2+^ overload [102]. Although the mitochondria possess the ability to accumulate substantial amounts of Ca^2+^ in their matrix, stockpiling high levels of Ca^2+^ has detrimental consequences, particularly the collapse of energy homeostasis [103]. Disturbances in mitochondrial Ca^2+^ handling are linked to neurodegenerative diseases such as Alzheimer's disease, Parkinson's disease and Huntington's disease [37, 104, 105]. Under conditions that disturb cytosolic Ca^2+^ homeostasis, the MCU rapidly load Ca^2+^ into the matrix at a rate that exceeds the mitochondrial sodium/calcium/lithium exchanger (NCLX) Ca^2+^ clearance [101]. This excessive rise in Ca^2+^ concentration in the mitochondria leads to increased permeability of the inner mitochondrial membrane (IMM), provoking IMM depolarisation and matrix swelling, eventually leading to compromised cell function and cell death [90, 104].

Ca^2+^ modulates the activity of TRP ion channels, which generates positive or negative feedback, moulding overall intracellular Ca^2+^ concentration [98]. As mentioned above, changes in the activity of ion channels and pumps that regulate Ca^2+^ have a central role in some disease onset and progression, including Alzheimer's, Parkinson's, Huntington's, and myopathies; hence, maintaining Ca^2+^ at a homeostatic balance is very important for optimal cell function [39, 61, 104, 105]. Given the vital role Ca^2+^ ions play in various important biological processes, more research focusing on exploring the potential implications of TRP channels and Ca^2+^ dysregulation to PCC onset and progression is required. The upstream disruptions, in this case, TRPM3 ion channel impairment in both PCC and ME/CFS [54], are believed to potentially lead to downstream disturbances in bioenergetics, such as the mitochondria, thus contributing to loss of cell activity. Accordingly, future investigations should investigate the functional consequences of impaired TRPM3 activity and associated downstream Ca^2+^ dysregulation in NK cells from individuals with PCC. In particular, it will be essential to determine the mechanistic link between TRPM3 dysfunction, Ca^2+^ signalling abnormalities, mitochondrial impairment, and reduced NK cell function reported in PCC. Given the central role of Ca^2+^ in regulating mitochondrial bioenergetics and immune cell effector functions, this may be further explored through assessment of mitochondrial reactive oxygen species (ROS) production, intracellular ATP levels, mitochondrial membrane potential, metabolic activity, and respiratory capacity using approaches such as Seahorse extracellular flux analysis.

The use of patient‐derived NK cells in this study enhances the biological and clinical relevance of the findings; however, the limited yield of primary cells per participant constrained the number of experimental conditions that could be performed. Moreover, in vitro expansion was not feasible due to the limited proliferative capacity of primary NK cells relative to immortalised lines, as prolonged culture is associated with cellular stress, phenotypic drift, senescence, and reduced viability, thereby limiting large‐scale expansion without compromising biological integrity.

This study used an extremely strict participant selection criterion; for instance, participants were excluded if diagnosed with other chronic illnesses or were extensively using medications that directly or indirectly influence TRPM3 or Ca^2+^ activity, hence our sample size was small. To mitigate this limitation, methodological steps were taken to increase the population of NK cells analysed per participant, strategically increasing the total number of cells analysed per group. Additionally, to mitigate potential technical limitations, protocol optimizations were performed, and six separate wells were imaged per participant, including single dye control wells. Further, previously optimized concentrations of PregS and ononetin were applied to limit potential off‐target effects. Inhibition of TRPM3 activity using ononetin, sequentially followed by activation using PregS, was performed to confirm TRPM3 channel activity. Further, to monitor and confirm mitochondrial uncoupling, FCCP was used at the end of each experiment.

In conclusion, research from this study provided new evidence that demonstrate that TRPM3 ion channel dysfunction has a significant impact on cytoplasmic and Ca^2+^ mobilization in the mitochondria in PCC. In addition, this study highlighted potential overcompensatory mechanisms in the mitochondria, possibly arising from altered Ca^2+^ signalling processes. Altered Ca^2+^ signalling can lead to broader systemic issues, impacting both the immune system and bioenergetic processes. Collectively, these findings provide an avenue for further studies on the physiological functions of the TRPM3 ion channel and its role in PCC and similar chronic illnesses. Further, the combination of electrophysiology and Ca^2+^ imaging protocols provides a comprehensive characterisation and analysis of Ca^2+^ influx via TRPM3 in NK cells of PCC and similar illnesses, such as ME/CFS patients. Recently, research has reported considerable overlap in symptom presentations predominantly associated with neurological, metabolic, and immunological disturbances between PCC and ME/CFS patients. In addition, a proportion of PCC patients meet the diagnostic criteria for ME/CFS. For this reason, it is crucial to employ a multidisciplinary approach for the investigation of both conditions. Further, considering both conditions have a significant impact on quality of life and an enormous impact on public health, more research focusing on understanding the pathomechanisms involved is required.

## Author Contributions

C.T.M. performed all the experiments, data analysis and writing of the manuscript. S.M.‐G. conceived the project and study coordination. C.T.M., N.E.‐F., K.M., and S.M.‐G. contributed to the experimental design. C.T.M., N.E.‐F., K.M., and S.M.‐G. contributed to the critical analysis of the data. All the authors critically reviewed this manuscript and approved the final version.

## Resource Availability

Requests for further information and resources should be directed to and will be fulfilled by the lead contact, Chandi Tabeth Magawa (chandi.magawa@griffithuni.edu.au; c.magawa@griffith.edu.au). This study did not generate new, unique reagents.

## Ethics Approval and Consent to Participate

This project was approved by Griffith University Human Research Ethics Committee (GU: 2022/666). All participants provided written consent before participation.

## Consent for Publication

All participants provided informed and written consent before participation.

## Conflicts of Interest

The authors declare no conflicts of interest.

## Supporting information




**Supporting File**: eji70240‐sup‐0001‐SuppMat.pdf.

## Data Availability

All relevant data are present within the manuscript. The datasets from this study are not publicly available due to confidentiality agreements, but could be available on reasonable request. Any request should be submitted to the Chair, Griffith University Human Research Ethics Committee, by email at research-ethics@griffith.edu.au.
