# Supplementary material for: Altered TRPM3‐Dependent Cytosolic and Mitochondrial Calcium Influx in Natural Killer Cells of Post‐COVID‐19 Condition Patients
Source: Eur J Immunol. 2026 Jul 22;56(7):e70240. doi: 10.1002/eji.70240 (PMC13390657; doi:10.1002/eji.70240)
Supplement: Supplementary file 1 — Supporting File: eji70240‐sup‐0001‐SuppMat.pdf. [file EJI-56-e70240-s001.pdf]

Supporting Information

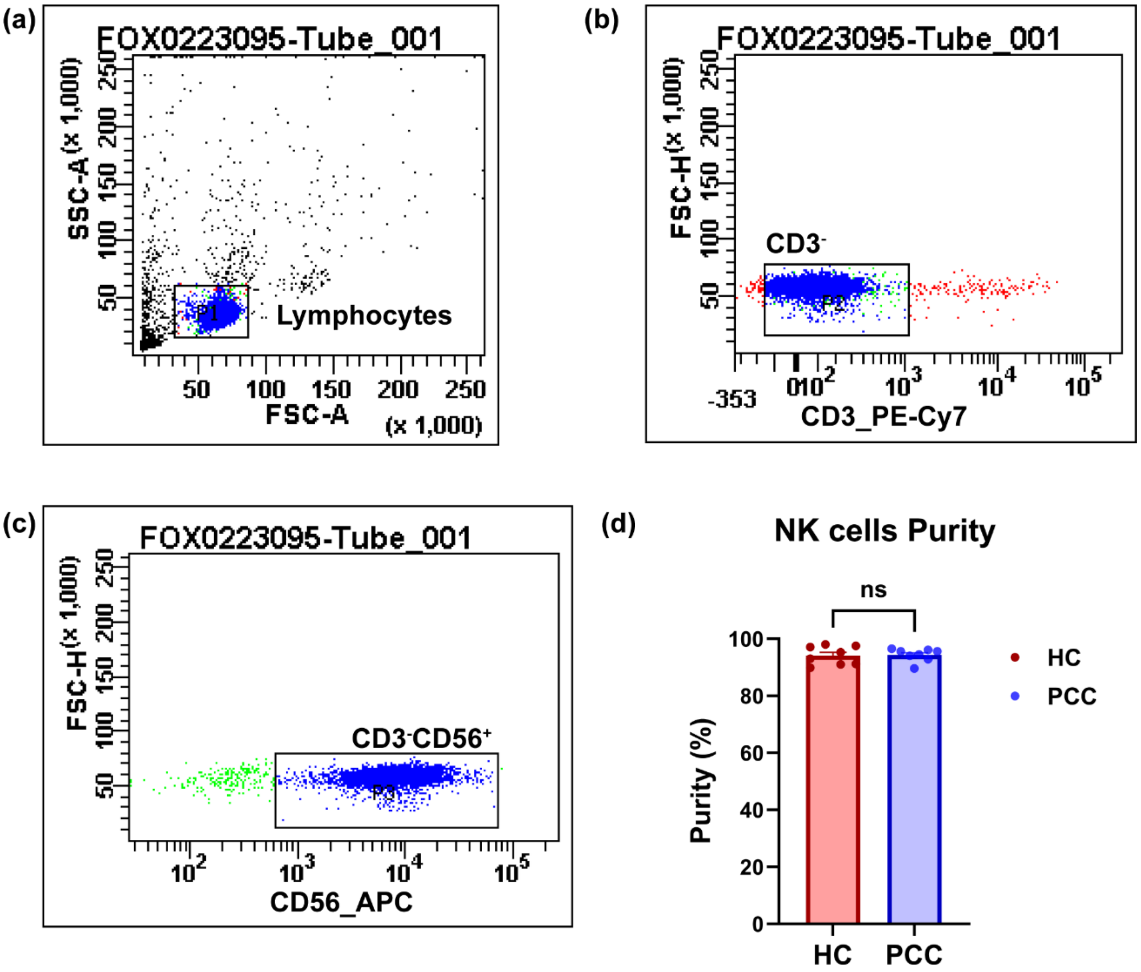

Figure S1: Natural Killer cells purity. Data are presented as mean ± SEM. The purity for NK cells ($CD3^{-}$ $CD56^{+}$) was 94.4 ± 0.80 % for PCC and 94.1 ± 1.15 % for HC as determined by flow cytometry. Cells were acquired at 10,000 events using the BD X20 flow cytometer (BD Biosciences, San Diego, CA, USA), using the following gating strategy: (A) Lymphocytes were gated using the SSC-A and FSC-A. (B) CD3 negative cells were selected from the lymphocyte population. (C) NK cells were identified as the CD3 negative and CD56 positive population. (D) Bar graphs representing NK cell purity (%). Abbreviations: NK, natural killer cells; PCC, post-COVID-19 condition; HC, healthy control; SEM, standard error of mean.

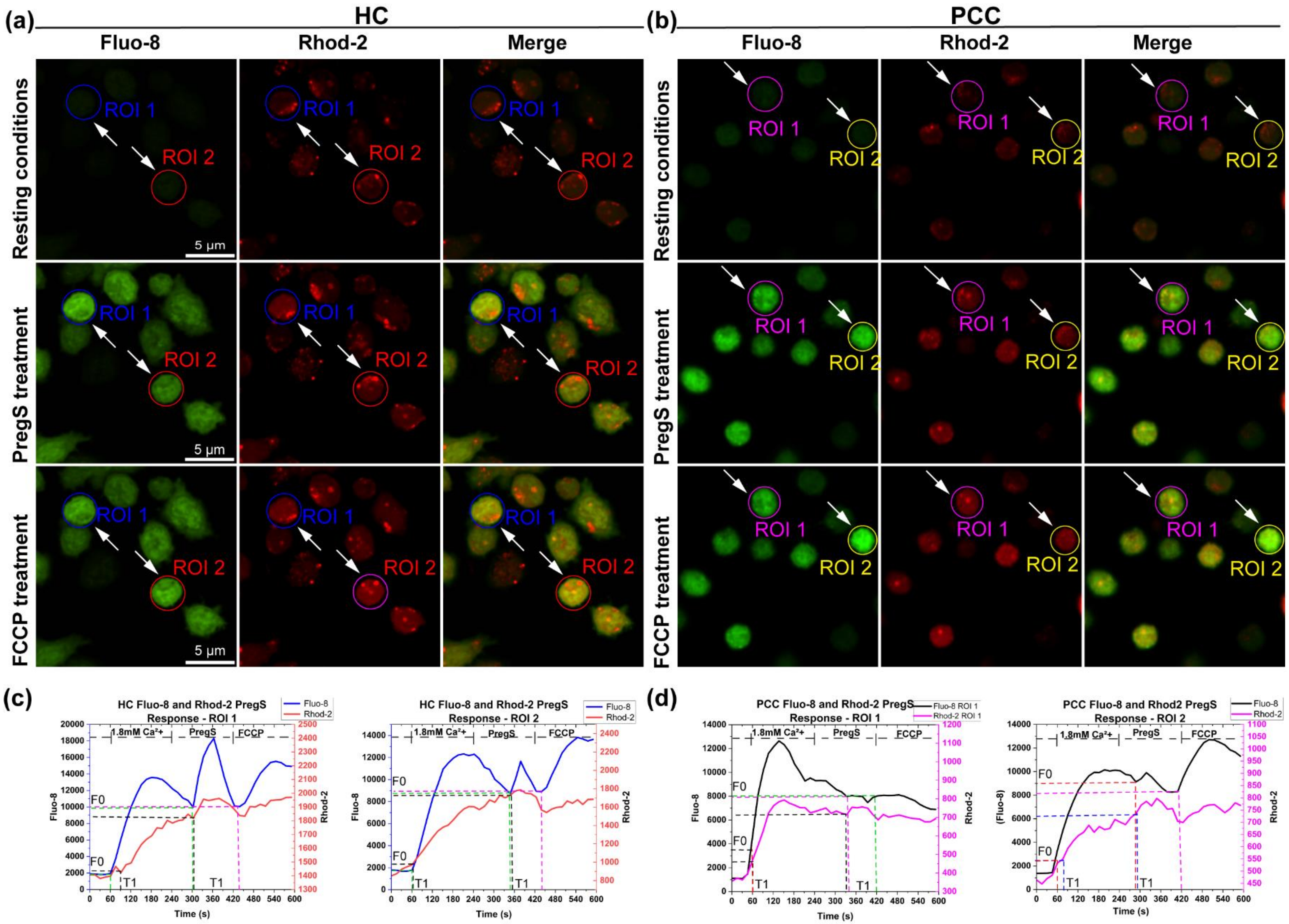
(a)
HC
Fluo-8
Rhod-2
Merge
(b)
PCC
Fluo-8
Rhod-2
Merge
Resting conditions
PregS treatment
FCCP treatment
ROI 1
ROI 2
5 µm
(c)
HC Fluo-8 and Rhod-2 PregS Response - ROI 1
HC Fluo-8 and Rhod-2 PregS Response - ROI 2
(d)
PCC Fluo-8 and Rhod-2 PregS Response - ROI 1
PCC Fluo-8 and Rhod2 PregS Response - ROI 2
Fluo-8
Rhod-2
Fluo-8 ROI 1
Rhod-2 ROI 1
1.8mM Ca²+
PregS
FCCP
F0
T1
Time (s)
(Fluo-8)

Figure S2: Simultaneous assessment of Ca^{2+} influx into the cytoplasm and mitochondria in NK cells. NK cells stained with Fluo-8 and Rhod-2 were imaged simultaneously confirming downstream TRPM3 -dependent Ca^{2+} mobilisation into the mitochondria. (A & B) NK cells from a HC participant and PCC patient, respectively stained with Fluo-8 and Rhod-2. (C) Fluorescence intensity curves for ROIs illustrated in A. (D) Fluorescence intensity curves for ROIs illustrated in B. The dotted lines indicate F0s and corresponding T1s for each stimulation condition. Abbreviations: Ca^{2+}, calcium; HC, healthy control; PCC, post-COVID-19 condition; ROI, region of interest; 4-(FCCP, Carbonyl cyanide trifluoromethoxy) phenylhydrazone; Fmax, maximum fluorescence; F0, minimum fluorescence; PregS, Pregnenolone sulphate. Imaging analysis was performed using cellSens Dimension Desktop by drawing perimeters around ROIs, then edited using Adobe illustrator. Time lapse fluorescence Graphs were constructed using Origin software.

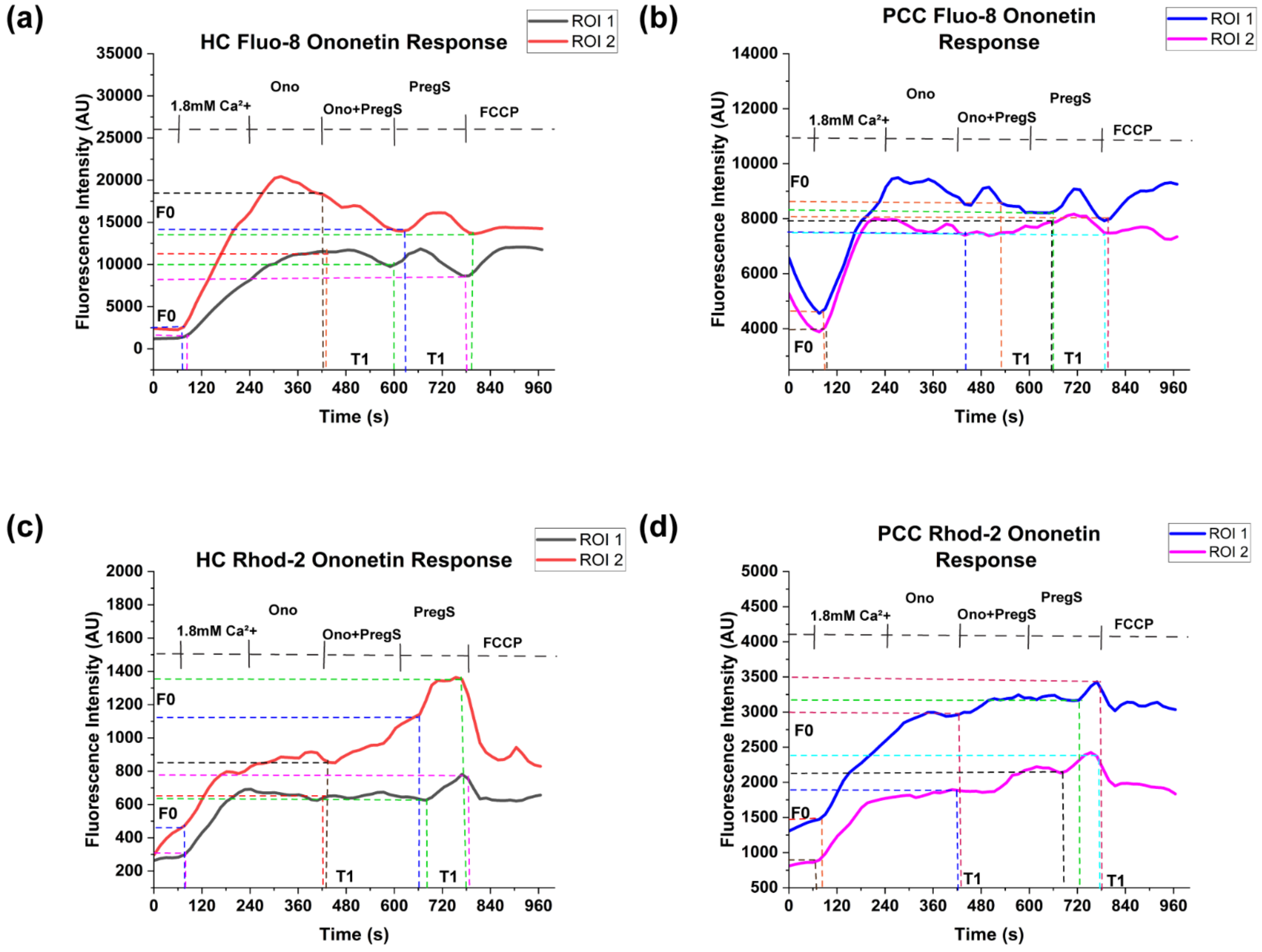
(a)
HC Fluo-8 Ononetin Response
ROI 1
ROI 2
1.8mM Ca²+
Ono
Ono+PregS
PregS
FCCP
F0
F0
T1
T1
Fluorescence Intensity (AU)
Time (s)
(b)
PCC Fluo-8 Ononetin Response
ROI 1
ROI 2
1.8mM Ca²+
Ono
Ono+PregS
PregS
FCCP
F0
F0
T1
T1
Fluorescence Intensity (AU)
Time (s)
(c)
HC Rhod-2 Ononetin Response
ROI 1
ROI 2
1.8mM Ca²+
Ono
Ono+PregS
PregS
FCCP
F0
F0
T1
T1
Fluorescence Intensity (AU)
Time (s)
(d)
PCC Rhod-2 Ononetin Response
ROI 1
ROI 2
1.8mM Ca²+
Ono
Ono+PregS
PregS
FCCP
F0
F0
T1
T1
Fluorescence Intensity (AU)
Time (s)

Figure S3: Modulation of TRPM3 with ononetin in NK cells. To confirm the activation of the TRPM3 channel by PregS, we applied the specific TRPM3 blocker ononetin along with PregS. The baseline was recorded in 0mM Ca^{2+} for 60s, sequentially followed by addition of 1.8 mM Ca^{2+}, inhibition by Ononetin alone, combined modulation using PregS and Ononetin, TRPM3 activation using PregS alone and ending the experiment with mitochondrial uncoupling using 1 µM FCCP. A decrement of the responses was observed upon addition of ononetin. (a & b) Examples of Fluo-8 time-lapse fluorescence intensity curves for ononetin response in NK cells from HC and PCC, respectively; (c & d) Rhod-2 time-lapse fluorescence intensity curves for ononetin response in NK cells from HC and PCC, respectively. The dotted lines indicate F0s and corresponding T1s for each stimulation condition. Abbreviations: Ca^{2+}, calcium; TRPM3, transient receptor potential melastatin 3 (TRPM3); PregS, Pregnenolone sulphate; PCC, post-COVID-19 condition; HC, healthy control; ROI, region of interest; Fmax, maximum fluorescence; F0, minimum fluorescence. Imaging analysis was performed using cellSens Dimension Desktop by drawing perimeters around ROIs, then edited using Adobe illustrator. Time lapse fluorescence Graphs were constructed using Origin software.

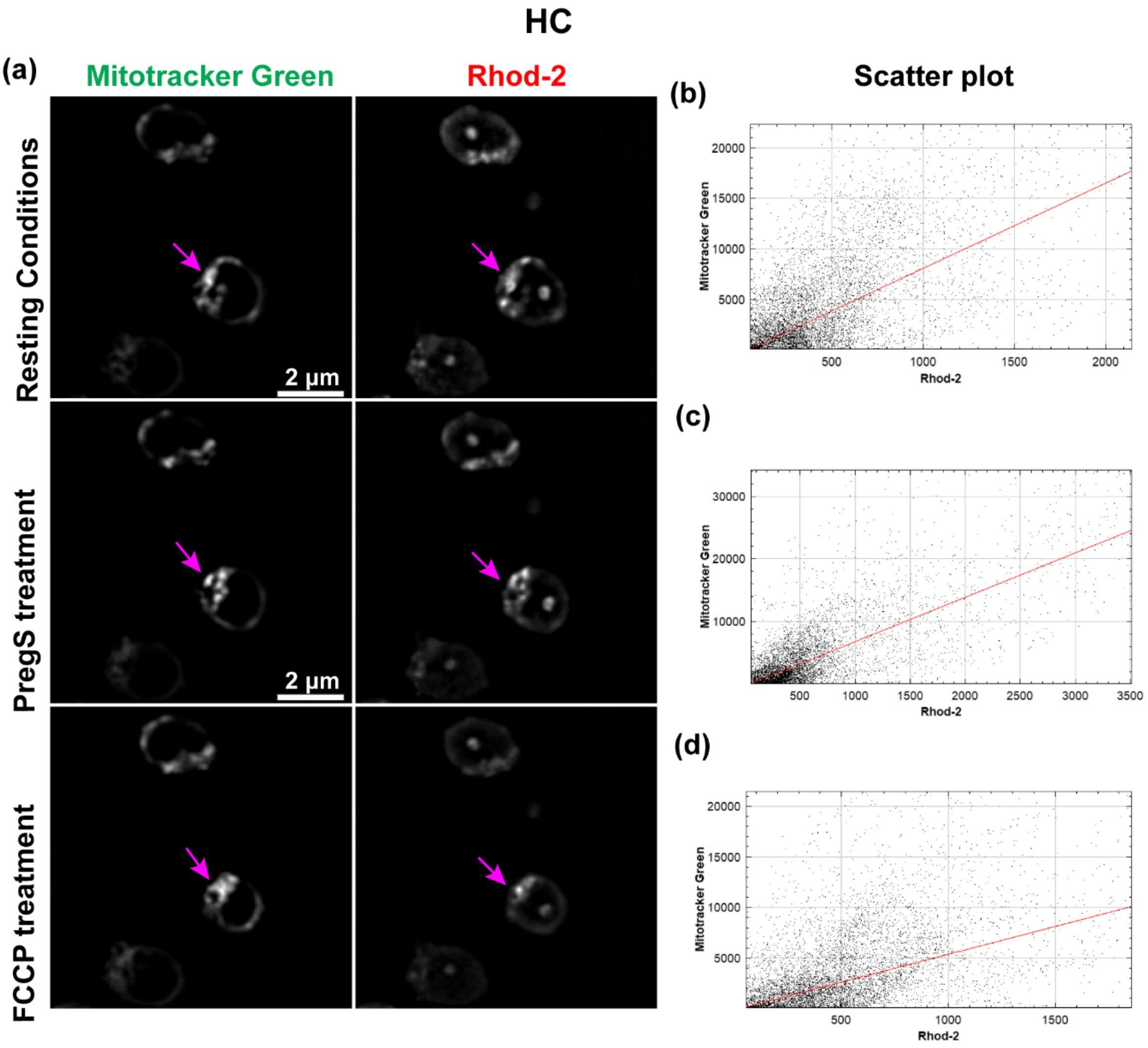

(e)

Coefficient	Resting Condition	PregS Treatment	FCCP Treatment
r	0.718	0.804	0.69
M1	0.759	0.808	0.767
M2	0.462	0.655	0.528

Figure S4: Colocalization of Mitotracker green and Rhod-2 signals Over time in NK Cells from a HC. (a) Representative grey scale images of NK cells from a HC stained with Mitotracker Green FM and Rhod-2 AM exhibiting similar changes in Rhod-2 fluorescence intensity over time, at 60 x. Arrows indicate mitochondrial networks in one cell containing both stains, showing change in fluorescence intensity overtime. Scatter plots (b-d) correspond to the colocalization of Mitotracker Green FM and Rhod-2 in response to changes in Ca^{2+} influx in the mitochondria. (b) Corresponding scatterplot of Mitotracker green and Rhod-2 fluorescence intensities

of NK cells at resting conditions. (c) Corresponding scatterplot of Mitotracker green and Rhod 2 fluorescence intensities of NK cells after stimulation with PregS, showing increased Rhod-2 fluorescence intensity, indicating Ca^{2+} mobilisation into the mitochondria, consequently, change in slope by the fitted line and rotation of the dotted cloud and towards Mitotracker Green axis. (d) Corresponding scatterplot of Mitotracker green and Rhod 2 fluorescence intensities of NK cells after treatment with FCCP showing decreased Rhod-2 fluorescence intensity as Ca^{2+} efflux the mitochondria into the cytoplasm, resulting in change of the slope towards Rhod-2 axis and separation of the dotted cloud. (e) Summary of the Pearson's r and Manders' coefficients, quantifying the degree of linear association and spatial overlap between fluorescence signals, respectively. Abbreviations: Ca^{2+}, calcium; Pearson's r, Pearson Correlation Coefficient; HC, Healthy Control; M1 = Rhod-2 and M2 = Mitotracker Green, MCC, Manders Correlation Coefficients; PregS, Pregnenolone sulphate; 4- (FCCP, Carbonyl cyanide trifluoromethoxy) phenylhydrazone.
